# Chemokine CCL17 Affects Local Immune Infiltration Characteristics and Early Prognosis Value of Lung Adenocarcinoma

**DOI:** 10.3389/fcell.2022.816927

**Published:** 2022-03-07

**Authors:** Ting Ye, Xuefang Zhang, Yongjian Dong, Jing Liu, Wenfeng Zhang, Fenglin Wu, Huaben Bo, Hongwei Shao, Rongxin Zhang, Han Shen

**Affiliations:** ^1^ Guangdong Provincial Key Laboratory of Biotechnology Candidate Drug Research, School of Life Sciences and Biopharmaceutics, Guangdong Pharmaceutical University, Guangzhou, China; ^2^ Department of Radiation Oncology, Dongguan People’s Hospital, Affiliated Dongguan Hospital of Southern Medical University, Dongguan, China

**Keywords:** CCL17, chemokine, tumor microenvironment, lung adenocarcinoma, immune cells

## Abstract

CCL17 is an important chemokine that plays a vital immunomodulatory role in the tumor microenvironment (TME). Analysis of lung adenocarcinoma (LUAD) data in Kaplan–Meier plotter databases found that the overall survival of patients in the CCL17 high-expression group was higher than that of the low-expression group, especially for patients with early (stages I and II) LUAD, which has a more positive prognostic value. Expression of CCL17 in LUAD was positively correlated with the proportion of tumor-infiltrating lymphocytes, immunostimulators, and major histocompatibility complexes using the TISIDB databases. Based on the RNA-seq and clinical data of 491 LUAD patients obtained from the TCGA database, 1,455 differential genes were found between the CCL17 high- and low-expression groups. Using WGCNA analysis confirmed that the expression of differential genes in the blue module is negatively correlated with poor survival and clinical stages of LUAD patients, and *CCL17* and *CCR4* genes belong to the hub genes in the blue module. Further analysis by the ESTIMATE and CIBERSORT algorithm found that the naive B cells and CD8^+^ T cells in the CCL17 high-expression group have a higher distribution ratio in the early LUAD patients, and the high immune score has a positive relationship with the overall survival rate. Using somatic mutation data of TCGA-LUAD, we found that 1) the tumor mutation burden values of the CCL17 high-expression group were significantly lower than those of the CCL17 low-expression group and 2) the expression levels of CCL17 and the tumor mutation burden values were negatively correlated. Transwell chemotaxis and cytotoxicity assays confirmed that CCL17 contributes to the migration of CCR4-positive lymphocytes into the H1993 LUAD TME and enhances the specific lysis of LUAD cells. In summary, high expression of CCL17 in the LUAD TME promotes local immune cell infiltration and antitumor immune response, which may contribute to the better survival and prognosis of patients with early LUAD.

## Introduction

The tumor microenvironment (TME) is the internal environment in which tumor cells produce and live (Hanahan and Coussens, 2012). The TME includes not only the tumor cells but also the fibroblasts, immune and inflammatory cells, glial cells, and other cells around them (Mlecnik et al., 2018), of which chemokines are an important category (Gregorio et al., 2018).

Chemokines are a group of small-molecule-secreted proteins that have chemotactic effects and a variety of biological effects on different target cells. Currently, more than 60 species of chemokines have been found, which can be divided into four subfamilies according to their N-terminal cysteine (C) arrangement. Among them, CCL17 belongs to the CC subfamily and plays an effective role in chemotaxis of CCR4+ T lymphocytes through the CCR4 receptor molecule (Viola et al., 2012). When chemokines interact with chemokine receptors, different immune cell subsets can be recruited into the TME, and these cell subsets play an important role in tumor development and therapeutic prognosis (Yoshie and Matsushima, 2015).

High expression of CCL17 in the TME of patients with gastric cancer (Mizukami et al., 2008) and Hodgkin’s lymphoma (Ishida et al., 2006) recruits large numbers of CD4^+^ Treg cells to infiltrate tumor tissues and inhibits CD8^+^ cytotoxic T lymphocyte function, resulting in local cellular immune suppression, and leading to a poor prognosis in patients. However, other investigators and our team have found that using mouse models of melanoma (Huang et al., 2021), colon cancer (Kanagawa et al., 2007), and pancreatic cancer (Zhan et al., 2017) increased CCL17 expression, induced local infiltration of CD8^+^ T cells into the tumor, and exerted antitumor effects.

According to the latest cancer statistics, although the incidence of lung cancer has declined, it is still the cancer with the highest mortality in the world (Sung et al., 2021). Among them, non-small cell lung cancer (NSCLC) accounts for 85% of all cases of lung cancer, and lung squamous cell carcinoma (LUSC) and lung adenocarcinoma (LUAD) are the main pathological types of NSCLC (Mao et al., 2019).

High expression of CCL17 in local tumors of NSCLC was found to be an important cause of Treg chemotaxis to tumor localization, causing suppression of the immune response (de Chaisemartin et al., 2011; Liu et al., 2017). Qin et al. showed that in lung cancer patients with malignant pleural effusions, CCL17 did not exert a chemotactic effect on Treg (Qin et al., 2009). Thus, the role of CCL17 in the microenvironment of NSCLC remains unclear and controversial.

In this study, we used public databases for bioinformatics analysis and found that CCL17 has significant prognostic value for LUAD patients. We analyzed the relationship between CCL17 expression and local immune cells and immune molecules in LUAD tumors through the TISIDB database. Subsequently, to analyze the impact of CCL17 expression levels on LUAD, we screened for differential TCGA-LUAD genes according to the grouping of high- and low-CCL17 expression levels and performed WGCNA analysis. In addition, ESTIMATE and CIBERSORT algorithms were used to provide insight into how CCL17 affects immune infiltration characteristics in the TME. The tumor mutation burden (TMB) values of CCL17 in LUAD were also analyzed using somatic cell mutation data. Finally, the effect of CCL17 on local immune cell infiltration of LUAD cells was verified by transwell chemotaxis and cytotoxicity assays. This study provides a useful reference for understanding the intrinsic relationship and molecular mechanisms of CCL17 in LUAD.

## Materials and Methods

**FIGURE 1:**
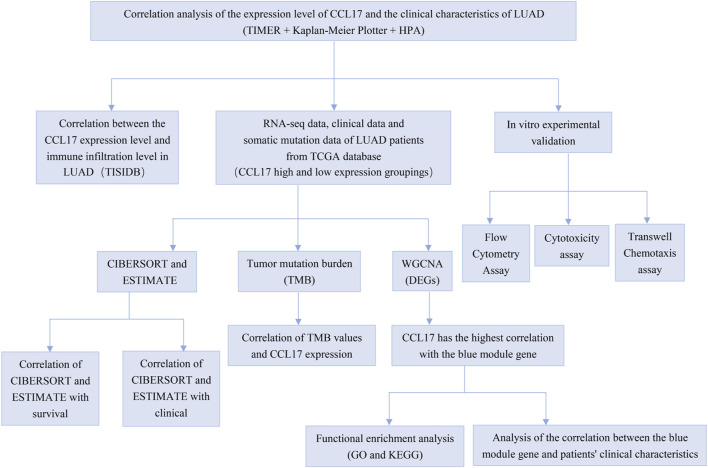
Flow chart for whole process analysis of articles.

The analysis details are presented in Figure 1.

### The mRNA Expression Level of CCL17 in Different Types of NSCLC in TIMER

The Tumor Immunity Estimation Resource (TIMER) database contains 10,897 samples of 32 cancer types from The Cancer Genome Atlas (TCGA). The differential expression of CCL17 between tumors and normal tissues can be explored by TCGA (The Cancer Genome Atlas) database (Li et al., 2017). The Wilcoxon test was used to determine the significance of the difference of results, and *p* < 0.05 was considered to be significant.

### The Survival Analysis of CCL17 in the Kaplan–Meier Plotter Database

The Kaplan–Meier plotter database was used to analyze the correlation between the expression of CCL17 in lung cancer and survival. The Kaplan–Meier database can evaluate the effect of the *54k* gene (mRNA, miRNA, and protein) on the survival rate of 21 cancer types. The sources of databases include GEO, EGA, and TCGA. We analyzed the correlation between the expression of CCL17 in lung cancer and overall survival (OS) and progression-free survival (PFS). The hazard ratio (HR) and logarithmic rank *p*-value with 95% confidence interval (CI) were calculated (Lanczky et al., 2016).

### Correlation Between Immune Cells and CCL17 Expression at the Overall Level in the TISIDB Database

TISIDB is a comprehensive database of tumors and the immune system used to analyze the correlation between tumors and 28 types of tumor-infiltrating lymphocytes (TILs),immunostimulators, and immune histocompatibility complexes (MHCs) (Ru et al., 2019). TISIDB allows users to explore the function of genes and their role in tumor–immune interactions. Spearman tests are used to measure the correlation between prognostic genes and TILs. Hypothesis tests were all two-sided, and *p*-values < 0.05 were considered statistically significant differences.

### Differentially Expressed Genes Screening

RNA-seq data and corresponding clinical information of LUAD patients were obtained from the TCGA data portal (https://tcga-data.nci.nih.gov/tcga/) and UCSC Xena database (https://xenabrowser.net/datapages/). The inclusion criteria of TCGA-LUAD samples are as follows: 1) providing the dataset of the LUAD gene expression profile; and 2) clinical data of LUAD patients are needed, including gender, age, clinical stages, and survival time. Finally, a total of 491 LUAD patients participated in the study.

Based on the survival time and survival status of 491 samples and the FPKM expression of CCL17 in the RNA-seq data, the CCL17 cut-off points were determined using the X-tile program (version 3.6.1; Yale, New Haven, USA), which identified the cut-off with the minimum *p* values from the log-rank chi2 statistics for the categorical CCL17 in terms of survival. According to their optimal cut-off value of 2.422, the samples were divided into 152 high-expression and 339 low-expression groups. The groupings performed for the subsequent analysis in this article were assigned according to this ratio, and FPKM expression values were used for the analysis. Then, the raw counts expression matrix of RNA-seq data from these 491 LUAD patients, after data preprocessing and quality assessment, we used two R packages “edgeR” and “DEseq2″ to screen DEGs between CCL17 high- and low-expression groups. We set *p* < 0.05 and |log_2_(FC)|>1 as the cut-off to select genes for further consideration in network construction.

### Establishment of the WGCNA Method

Weighted correlation network analysis (WGCNA) is a typical systems biology algorithm for constructing gene expression networks, capable of dividing gene co-expression networks of complex biological processes into several highly correlated feature modules, which represent several sets of highly synergistic variation of gene sets. The data matrix of gene expression in TCGA-LUAD was constructed, and the differentially expressed genes between CCL17 high- and low-expression groups were selected as the input dataset of the subsequent WGCNA package used in R/Bioconductor software (version: 3.6.3). An appropriate soft threshold power was selected to ensure a scale-free network. Then, the gene dendrogram and module identification were completed with dynamic tree cutting by the construction of adjacency and topological overlap matrix (TOM) and the calculation of the corresponding phase dissimilarity, and the minimum module size was 20 (Wang and Yang, 2020). The dynamic tree cut method used in the WGCNA analysis is performed using the “cutreeDynamic” function package. The topological overlap matrix (TOM) reflects the relative interconnectivity between two genes based on their degree of shared neighbors across the whole network (Chen et al., 2017). Finally, the correlation heat map between modules and traits was drawn to determine the correlation between modules of CCL17 and patients’ clinical characteristics (survival time, survival status, age, gender, and clinical stages).

### GO and KEGG

To elucidate the biological functions of blue module DEGs, we used the R software “clusterProfiler” package to conduct Gene Ontology (GO) enrichment analysis and Kyoto Encyclopedia of Genes and Genomes (KEGG) pathway analysis on blue module DEGs. GO functional enrichment analysis divides gene function into three components: cellular component (CC), molecular function (MF), and biological process (BP). The results of GO enrichment and the KEGG pathway are shown as bubble plots with *p*-value <0.05 as the threshold value.

### Stromal and Immune Scores of CCL17 High-and Low-Expression Groups

The stromal score and immune score in the TCGA-LUAD dataset were calculated using the ESTIMATE package of R software to estimate tumor purity for each LUAD sample. The “filterCommonGenes” and “estimatescore” functions were used to select the genes used by the ESTIMATE algorithm and used the expression levels of these genes to calculate an ESTIMATE score for each sample. For TCGA-LUAD samples, correlations between stromal/immune score and clinical characteristics were analyzed. The prognostic value of the stromal score and immune score was evaluated by the Kaplan–Meier method with the log-rank test (Ren et al., 2020).

### Relationship Between 22 TIIC Subtypes and CCL17 High-and Low-Expression Groups

Normalized gene expression data of the TCGA-LUAD dataset were used to infer the relative proportions of 22 TIIC subtypes using the CIBERSORT package of R software. For each sample, the sum of all estimated values of immune cell type components is equal to 1, and only patients with CIBERSORT *p* < 0.05 are considered eligible for further analysis (Zhang et al., 2020). According to CCL17 gene expression of LUAD patients, the ratios of TIICs in a high-expression group and low-expression group were compared, and its influence on the differential expression of 22 kinds of immune cells and the content proportion of 22 immune cells in the CCL17 high-expression group were analyzed. At the same time, the correlation between the OS and clinical stage of TCGA-LUAD and 22 kinds of immune cells was further analyzed, with *p* < 0.05 considered statistically significant.

### Analysis of TMB of CCL17 in LUAD

The somatic mutation data of TCGA-LUAD were obtained from the TCGA database (https://portal.gdc.cancer.gov/). We selected the “Masked Somatic Mutation” data processed based on VarScan software and performed the visualization process using the “maftools” R package with several analysis modules (Zhang et al., 2019). The TMB is the total number of mutations per megabase in the tumor tissue. In our study, we used a Perl script (JAVA8) to calculate the number of somatic variants/effective coverage area detected per sample. Finally, the differences in TMB values between the two groups were compared according to the CCL17 high-and low-expression groupings. Mutation information for CCL17 was downloaded from the cBioPortal database (https://www.cbioportal.org/) (Cerami et al., 2012; Gao et al., 2013).

### Analysis of Protein Expression Levels of CCL17 and Immune Cell Marker Molecules in the HPA Database

The Human Protein Atlas (HPA) database (https://www.proteinatlas.org) is a public database that provides millions of immunohistochemical images of the tissue and cellular distribution of human proteins (Uhlén et al., 2015). This study used immunohistochemical (IHC) staining from the HPA database to analyze the protein expression levels of CCL17 and immune cell marker molecules in LUAD tissue.

### Cell Culture and Flow Cytometry Assay

Human peripheral blood was collected from HLA-A2 healthy volunteers. Whole blood, diluted with physiological saline, was added to the surface of lymphocyte separation solution and centrifuged at 4°C. The liquid in the tube was divided into four layers, and the mononuclear cell cloud (PBMC) in the second layer was aspirated, washed twice with saline, resuspended in serum-free medium, counted, and set aside. HLA-A2-positive LUAD H1993 cells in the logarithmic growth phase were taken, made into cell suspension with the RPMI-1640 medium (Gibco), repeatedly freeze-thawed (-80–37°C), and filtered and de-bacterized using a 0.22-um microporous filter membrane as a tumor freeze-thaw antigen, and protein concentration was determined by the BCA method. Adjust PBMC concentration to 5 × 10^6^/ml and tumor freeze-thaw antigen concentration to 100ug/ml (incubation for 2 weeks). The cells were collected into centrifuge tubes, ensuring 1 × 10^6^/ml per tube, and 2ul of PE fluorescent-labeled CCR4 monoclonal antibody (BD Biosciences) was added, blown gently and evenly, incubated for half an hour at 4°C in the dark, and sorted CCR4-positive cells using a MoFlo cytometer (Beckman Coulter). The selected CCR4-positive cells were added with anti-CD4-PECY5 mAb and anti-CD8-FITC mAb (BD Biosciences) and incubated for half an hour at 4°C in the dark, and then the ratio of CD4-positive and CD8-positive cells was detected by flow cytometry.

### Transwell Chemotaxis Assay

The chemotaxis assays were performed in Costar Transwell 24-well plates (Costar Corning), each well of which has a lower chamber and an upper chamber separated by a polycarbonate membrane with 5 microM pores. The CCR4-positive cell suspension (100ul, 2×10^6^ cells/ml) was added to the upper chamber of both treatment and control groups, 600ul of chemokine CCL17 (R&D systems) was added to the lower chamber of the treatment group, and an equal volume of the RPMI-1640 medium (Gibco) was added to the lower chamber of the control group. Chemotaxis was performed for 2 h, and the number of cells was counted in the lower wells after staining with CFSE.

### Cell Cytotoxicity Assay

HLA-A2-positive LUAD H1993 cells were incubated with calcein-AM (1 μm/L) for 30 min. After washing, a 1×10^5^ cells/ml tumor cell suspension was prepared. Transwell 24-well plates were taken, and A and B treatment groups, positive control group, and background control group were set up. The lower chambers of all four groups were inoculated with H1993 cells at a density of 1 × 10^4^ cells/ml, and 2% Triton X-100 with target cells was used as a positive control. After the cells in the lower chamber were opposed, the chemokines CCL17 (100 ng/ml) were added into the lower chambers of treatment group A, without addition of chemokines in treatment group B and background control group. The upper chambers of the treatment groups and the positive control group were added with 100 ul of CCR4-positive cell suspension (2×10^6^ cells/ml). An equal volume of the RPMI-1640 medium (100 ul) was added to the upper chamber of the background control group.

The cells were harvested for analysis 4 h later. Fluorescence intensity (FI) was measured using a Varioskan LUX instrument (Thermo Fisher Science). The specific lysis index was calculated as (FI treatment wells − FI background wells)/(FI positive wells − FI background wells).

### Statistical Analysis

Experimental data were analyzed with GraphPad Prism 7 (GraphPad software). All data were presented as mean ± SD. The experimental results were statistically analyzed for significant difference using the two-tailed Student’s t-test for two groups, and one-way analysis of variance (ANOVA) for more than two group analysis. The results were considered statistically significant at *p* < 0.05.

## Results

### The mRNA Expression Level of CCL17 in Different Types of NSCLC

**FIGURE 2:**
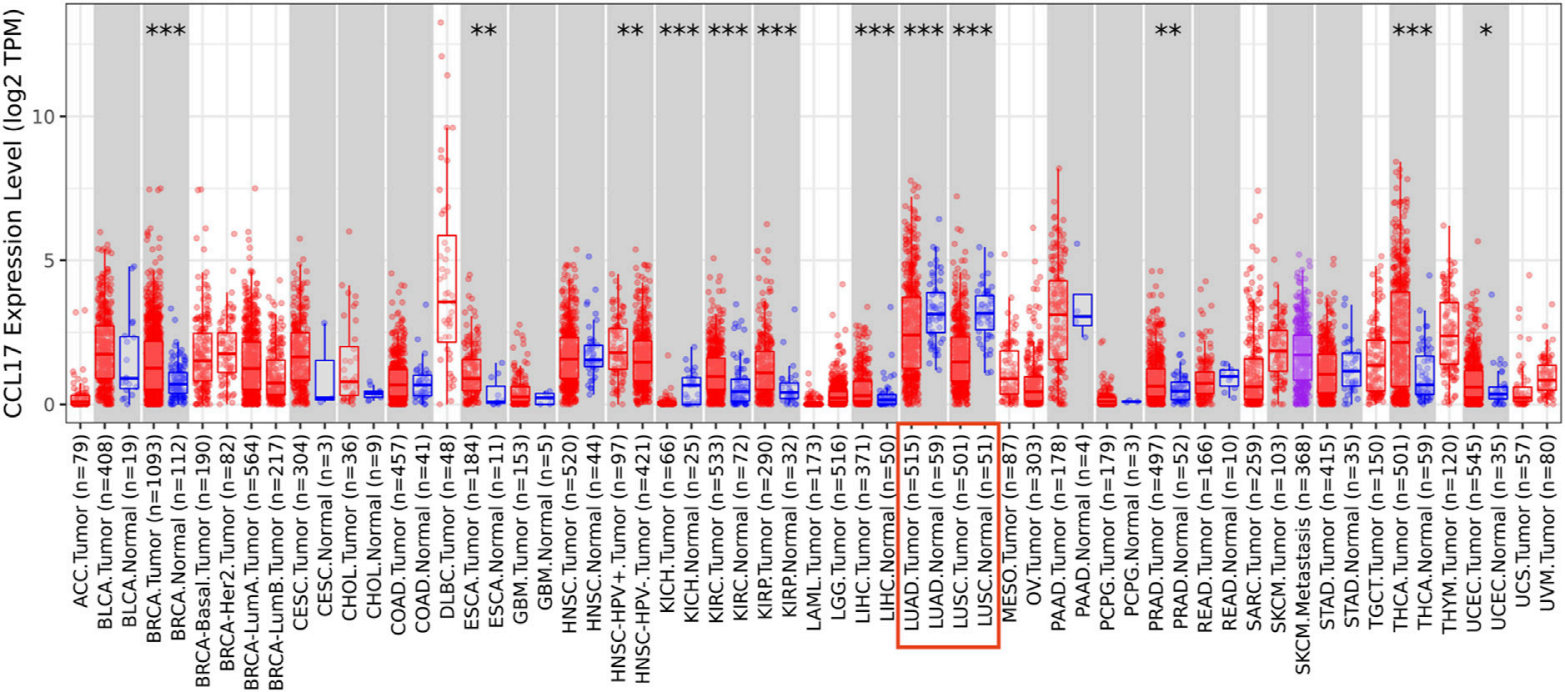
Difference of CCL17 expression levels in LUAD and LUSC in TIMER (*p < 0.05, **p < 0.01, ***p < 0.001).

The TIMER database was employed to analyze the differential expressions between tumor and adjacent normal tissues for CCL17 across all TCGA tumors. As shown in Figure 2, CCL17 expression was significantly decreased in both LUAD and LUSC tissues compared with adjacent normal tissues (*p* < 0.001).

### CCL17 High Expression Affects the Prognosis of LUAD

The relationship between expression levels of CCL17 and lung cancer patient survival was analyzed through the Kaplan–Meier plotter database.

With further studies on clinical characteristics of lung cancer patients, we found that CCL17 mainly affected the survival status of early-stage lung cancer patients. As shown in Table 1, CCL17 high expression was significantly correlated with good prognosis of lung cancer patients at stage 1 (OS, HR = 0.59 (0.45–0.78), *p* = 0.00018; PFS, HR = 0.55 (0.36–0.85), *p* = 0.0064) but did not significantly correlate with OS and PFS in patients with lung cancer at stage 2–4. Meanwhile, CCL17 high expression had a positive effect on the prognosis of lung cancer patients in T1 (OS, HR = 0.63 (0.46–0.86), *p* = 0.0029; PFS, HR = 0.39 (0.24–0.65),*p* = 0.0002), N0 (OS, HR = 0.73 (0.53–0.93), *p* = 0.012; PFS, HR = 0.7 (0.49–0.98),*p* = 0.0366), N1 (OS, HR = 0.72 (0.5–0.99), *p* = 0.0445), and M0 (OS, HR = 0.68 (0.54–0.87), *p* = 0.0015) but was not associated with OS and PFS in T2, T4, and N2 stages and PFS in N1 and M0 stages.

**TABLE 1:** Correlation between CCL17 expression level and prognosis in lung cancer with different clinicopathological features by Kaplan–Meier plotter.

Clinicopathological characteristics	overall survival (n = 1927)			progression-free survival (n = 982)		
	N	Hazard ratio	*P*-value	N	Hazard ratio	*P*-value
Sex						
** **Female	714	0.74(0.58–0.93)	0.01	468	0.65(0.49–0.87)	0.0032
** **Male	1100	0.74(0.63–0.87)	0.00018	514	0.72(0.56–0.93)	0.012
Stage						
1	577	0.59(0.45–0.78)	0.00018	325	0.55(0.36–0.85)	0.0064
2	244	0.85 (0.58–1.25)	0.41	130	1.43 (2.84–2.44)	0.1851
3	70	0.6 (0.33–1.1)	0.094	—	—	—
Stage T						
1	437	0.63(0.46–0.86)	0.0029	172	0.39(0.24–0.65)	0.0002
2	589	0.83 (0.65–1.06)	0.13	351	0.78 (0.55–1.1)	0.1476
3	81	2.15(1.26–3.66)	0.0042	21	0.56 (0.21–1.53)	0.2551
4	46	0.63 (0.34–1.18)	0.15	—	—	—
Stage N						
0	781	0.73(0.53–0.93)	0.012	374	0.7(0.49–0.98)	0.0366
1	252	0.72(0.5–0.99)	0.0445	130	0.72 (0.34–1.2)	0.2026
2	111	0.82 (0.52–1.28)	0.37	51	1.35 (0.65–2.82)	0.4235
Stage M						
0	681	0.68(0.54–0.87)	0.0015	195	0.73 (0.43–1.23)	0.2321
Histology						
Adenocarcinoma	719	0.68(0.54–0.86)	0.0011	461	0.59(0.43–0.82)	0.0012
Squamous cell carcinoma	524	0.86 (0.68–1.1)	0.23	141	1.26 (0.72–2.19)	0.4209
Smoking history						
Exclude those never smoked	820	0.79(0.63–0.99)	0.037	603	0.6(0.39–0.94)	0.0238
Only those never smoked	205	1.76(1–3.11)	0.0491	193	0.55(0.34–0.89)	0.0144

N category refers to lymph node involvement; N0 indicates no regional lymph node metastasis, and N1∼N3 indicate increased regional lymph node metastasis. T category refers to tumor size or degree of infiltration; T1∼T4 indicate an increase in tumor volume and an increase in the extent of adjacent tissue involvement. M category refers to distant metastasis (usually bloodstream metastasis); M0 indicates those without distant metastasis, and M1 indicates those with distant metastasis. On this basis, the combination of the three TNM indicators was used to delineate the specific clinicopathological stage (Wang et al., 2020).

Notably, in the tissue type classification, there were two pathological types of lung cancer, adenocarcinoma and squamous cell carcinoma, in which CCL17 high expression was strongly associated with good prognosis in adenocarcinoma (OS, HR = 0.77 (0.68–0.867), *p* = 4.9e-05; PFS, HR = 0.59 (0.43–0.82), *p* = 0.0012), while the relationship with the prognosis of squamous cell carcinoma was not significant (*p* > 0.05).

The results of the analysis showed that CCL17 was more accurate for determining the prognostic value of LUAD patients, especially in the early clinical stage of LUAD patients, and CCL17 high expression had a significant positive effect on the survival prognosis of patients. Therefore, we subsequently focused on the analysis of the relationship between the expression level of CCL17 and the clinical characteristics of LUAD patients.

### The Overall Expression Level of CCL17 Is Related to the Immune Infiltration Level of LUAD

CCL17 belongs to the chemokine family, an immune function-related gene, which may be closely related to the status of immune cells in the TME. Then, to validate the relationship between CCL17 expression and immune cell infiltration, the correlation between CCL17 expression and TILs, immunostimulators, and MHCs in LUAD patients was further investigated using the TISIDB database.

**FIGURE 3:**
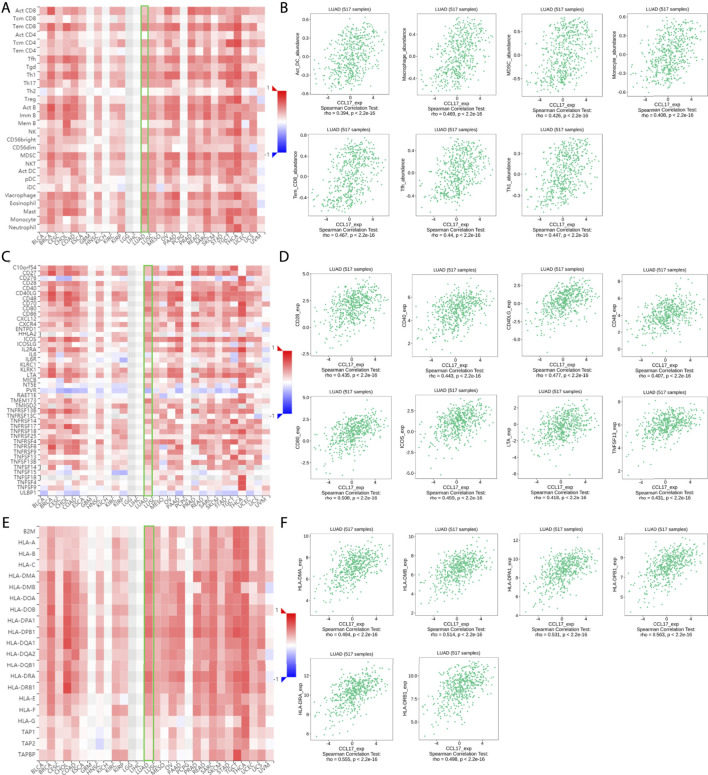
**(A-F)** Correlation of CCL17 with TILs, immunostimulators, and MHCs in TISIDB. **(A-B)** Correlation of CCL17 expression with 28 types of TILs in LUAD. **(C-D)** Correlation of CCL17 expression with immunostimulators in LUAD. **(E-F)** Correlation of CCL17 expression with MHCs in LUAD.

The expression of CCL17 was found to correlate significantly with the abundance of 28 different types of TILs (Figure 3A) and immunostimulators (Figure 3C) and MHCs (Figure 3E) in human malignancies by the TISIDB database, with a moderate-to-strong correlation (we used a correlation rho value of 0.4 as a criterion, and above 0.4 indicates a strong correlation).

Among them, CCL17 expression was positively correlated with the abundance of these types of TILs, such as (effector memory T cell) tem_CD8 cell (rho = 0.467, *p* < 0.001), (follicular helper T cell) Tfh (rho = 0.44, *p* < 0.001), (dendritic cell) Act DC (rho = 0.394, *p* < 0.001), macrophages (rho = 0.469, *p* < 0.001), (bone marrow-derived suppressor cell) MDSC (rho = 0.426, *p* < 0.001), Th1 (rho = 0.447, *p* < 0.001), and monocytes (rho = 0.408, *p* < 0.001) (Figure 3B).

A significant correlation was also found between CCL17 and most of the immunostimulators and MHCs. Among them, the expression of CCL17 was positively correlated with these immunostimulators related to B cell and T cell activation, such as CD28 (rho = 0.435, *p* < 0.001), CD40 (rho = 0.443, *p* < 0.001), CD48 (rho = 0.407, *p* < 0.001), CD40LG (rho = 0.477, *p* < 0.001), CD80 (rho = 0.508, *p* < 0.001), ICOS (rho = 0.459, *p* < 0.001), LTA (rho = 0.418, *p* < 0.001), and TNFSF13 (rho = 0.431, *p* < 0.001) (Figure 3D).

**FIGURE 4:**
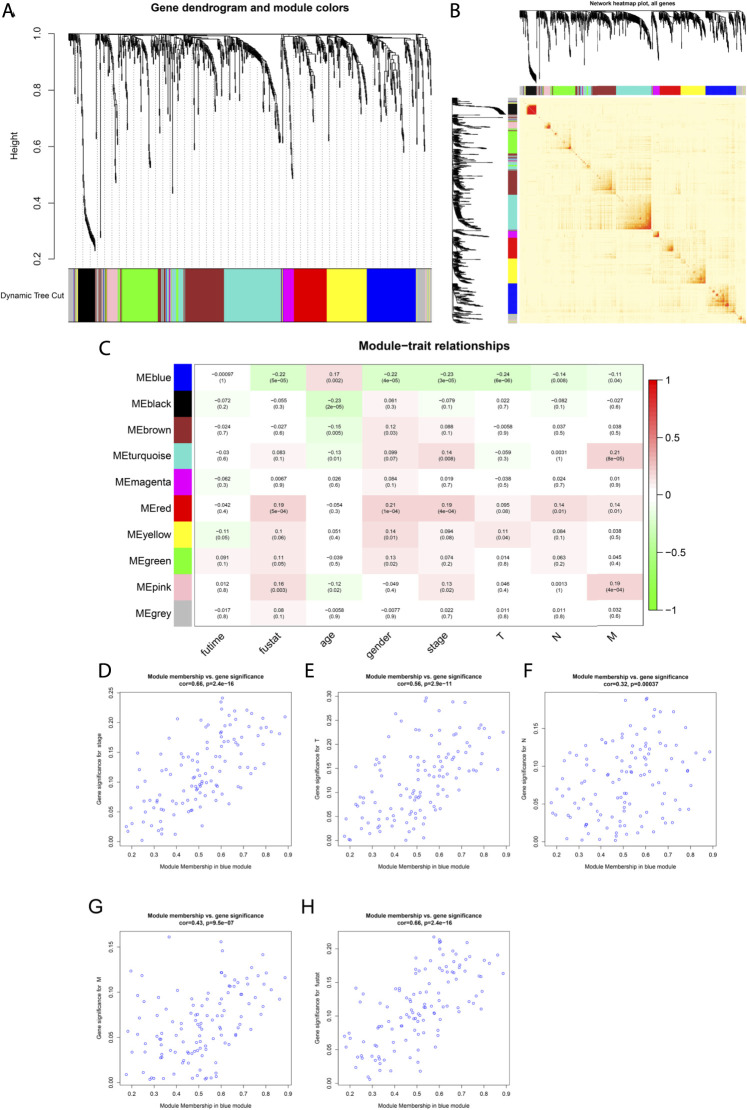
Weighted co-expression network construction and identification of the key module containing CCL17. **(A)** Cluster dendrogram was built based on the dissimilarity of the topological overlap, which presents ten gene co-expression modules in LUAD; the gray module indicates no co-expression between the genes. **(B)** Heat map describes the TOM among selected 1,455 genes in WGCNA, and the darker color represents higher overlap, and the lighter color corresponds to lower overlap. **(C)** Heat map of the correlation between module eigengenes and clinical traits of LUAD patients. **(D-H)** Scatter plot of GS for clinical stages, TNM stage, and fustat vs. MM in the blue module.

The expression of CCL17 was also positively correlated with MHCs located in specialized presenting cells such as dendritic cells, macrophages, and involved in antigen presentation as well as T cell differentiation, such as HLA-DPA1 (rho = 0.531, *p* < 0.001), HLA-DPB1 (rho = 0.563, *p* < 0.001), HLA-DMA (rho = 0.494, *p* < 0.001), HLA-DMB (rho = 0.514, *p* < 0.001), HLA-DRA (rho = 0.555, *p* < 0.001), and HLA-DRB1 (rho = 0.498, *p* < 0.001) (Figure 4F).

### The Blue Module Containing CCL17 Is Significantly Correlated With the Clinical Progress of LUAD

Based on the aforementioned analysis, it was found that the different expression levels of CCL17 in LUAD would have an impact on the survival prognosis of patients, and the underlying mechanism deserves further analysis and study.

A total of 1,455 DEGs were screened for differential analysis based on FDR<0.05 and |log_2_FC|>1, by grouping as described previously, and selected for subsequent WGCNA analysis. In addition, the differentially expressed genes (DEGs) intersected by the two differential analysis packages were summarized using Venn diagrams (Supplementary Figure S1).

Among the aforementioned 491 TCGA-LUAD samples, only 339 cases had complete survival time and clinicopathological stage (stage, TNM) data, so we extracted the clinical trait information of these 339 samples for the subsequent analysis of WGCNA. The power of *β* = 4 (scale freeβ^^2^ > 0.9) was selected as the soft-thresholding parameter to conduct a scale-free network (Supplementary Figure S2). In all, 10 co-expressed gene modules were identified by the dynamic tree cut method, and each of the modules was marked by a color (Figure 4A). The heat map plotted the TOM among 1,455 differentially expressed genes in the analysis, which revealed that every module was an independent validation to each other (Figure 4B).

As shown in Figure 4C, CCL17 is in the blue module, which significantly correlated with LUAD patients at stage (*R* ^2^ = -0.23, P = 3e-05), T stage (*R* ^2^ = −0.24, P = 6e −06), stage N (*R* ^2^ = −0.14, *p* = 0.008), stage M (*R* ^2^ = −0.11, *p* = 0.04), and survival status (fustat) (*R* ^2^ = −0.22, P = 5e −05). The blue module was identified as the key module for the highest correlations with the pathologic stage and survival status of LUAD patients and was negatively correlated. It indicates that with the decrease of CCL17 expression, the tumor stage increases, and the decrease of CCL17 expression is unfavorable to patient survival. Meanwhile, the correlation between gene significance (GS) and module membership (MM) in the blue modules was performed by scatter plot analysis. The horizontal coordinate value MM indicates the correlation of the gene with the module, and the vertical coordinate value GS indicates the correlation of the gene with the phenotypic trait. Generally, if GS and MM were highly associated, it implied that genes were the highly important elements for modules and were most significantly correlated with the trait. It is noteworthy that the blue module was associated with LUAD patients in stage (cor = 0.66, *p* = 2.4e-16), stage T (cor = 0.56, *p* = 2.9e-11), stage N (cor = 0.32, *p* = 0.00037), stage M (cor = 0.43, *p* = 9.5e-07), and survival status (fustat) (cor = 0.66, *p* = 2.4e-16) (Figures 4D–H).

The MM of CCL17 in the blue module is 0.760, and the cell membrane surface receptor CCR4, which is required for CCL17 chemotactic cells to undergo migration, also belongs to the blue module (MM = 0.601), indicating that both CCL17 and CCR4 are hub genes in the blue module.

### GO and KEGG Enrichment Analysis of Blue Module Genes

**FIGURE 5:**
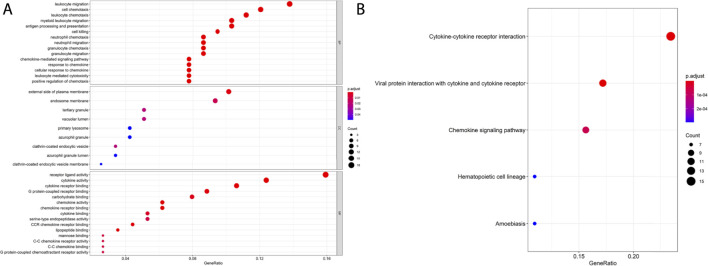
GO and KEGG enrichment analysis of blue module genes. **(A)** GO enrichment of the blue module gene. **(B)** KEGG pathway analysis of the blue module gene.

To elucidate the biological functions of blue module DEGs, we performed functional annotation and pathway enrichment analysis by using the “GO” and “KEGG” packages of R/Bioconator software and drew the bubble plot for GO analysis and KEGG pathway (*p* < 0.05).GO analysis is shown in Figure 5A, and its most important biological processes (BP) include “leukocyte migration,” “cell chemotaxis,” “lymphocyte migration,” and “antigen processing and presentation.” The cellular component (CC) consists mainly of the “external side of the plasma membrane,” “endosomal membrane,” and “tertiary granule.” Molecular functions (MF) are mainly enriched in “receptor–ligand activity,” “cytokine activity,” “cytokine receptor binding,” and “G protein-coupled receptor binding.” KEGG pathway analysis showed that the blue module genes play a key role in the cytokine–cytokine receptor interaction pathway, chemokine signaling pathway, and cancer pathway (Figure 5B).

### Comparison of Immune and Stromal Score Differences Between CCL17 High-and Low-Expression Groups in LUAD

Next, to compare the differences in survival status and clinical stages of LUAD patients in CCL17 high- and low-expression groups correlation with immune/stromal scores, immune and stromal scores were performed on TCGA-LUAD data using the ESTIMATE package of R software.

In the TME, immune cells and stromal cells are two main types of non-tumor components. The content of stromal cells and immune cells can be predicted by using the ESTIMATE algorithm, to judge the purity of the tumor (Bi et al., 2020). The samples from TCGA-LUAD were divided into CCL17 high- and low-expression groups based on the CCL17 mRNA-FPKM expression and scored for immunity and stroma, and the immune score was considered a tangible indicator of tumor prognosis. Higher estimate scores in immune scoring or stromal scoring represent large amounts of immune or stromal components in TME. The estimated value is the sum of the immune score and stroma score, indicating the combined proportion of both components in the TME.

The results showed that the immune and stromal scores were significantly higher in the CCL17 high-expression group than in the CCL17 low-expression group. The immune scores of LUAD patients in the CCL17 high-expression group ranged from 209.74 to 3,216.69, while the immune scores of patients in the CCL17 low-expression group ranged from -940.67 to 2,967.02. The mean immune score of the former (1,917.50) was significantly higher than that of the latter (1,278.45), implying that the CCL17 high-expression group among LUAD patients was more immune than the CCL17 low-expression group.

### Scores in the CCL17 High-Expression Group Were Significantly Associated With OS and Clinical Stages of LUAD

To determine whether the difference in CCL17 mRNA expression levels affects the correlation between the proportion of immune and stromal components in TME and OS in LUAD patients, Kaplan–Meier survival was performed to determine the potential correlations of OS with the immune and stromal scores.

**FIGURE 6:**
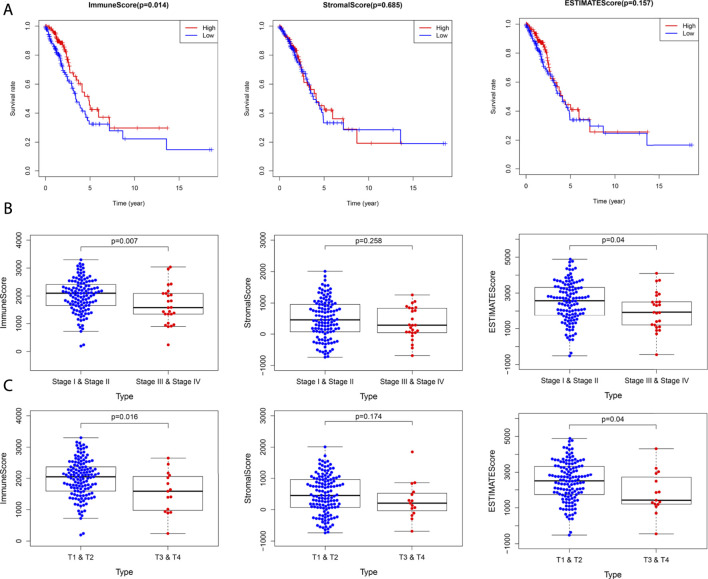
**(A)** Correlation between survival and score in LUAD patients in the CCL17 high-expression group. **(B)** Stage classification of the CCL17 high-expression group. **(C)** T classification of the CCL17 high-expression group.

As shown in Figure 6A, in the CCL17 high-expression group, the immune score was positively correlated with OS (*p* = 0.014), and patients in the high immune score group had a higher survival rate. In contrast, in the CCL17 low-expression group, none of the scores were significantly correlated with the OS (*p* > 0.05) (Supplementary Figure S3A). These results suggest that the immune component in TME in the CCL17 high-expression group contributes to the prognosis of LUAD patients.

Next, the correlation of immune and stromal component scores with clinical characteristics (stage classification and TNM stage) in LUAD patients in the CCL17 high- and low-expression groups was compared. The immune scores were negatively correlated with stage (*p* = 0.007) and stage T (*p* = 0.016) in the CCL17 high-expression group, while stromal scores were not statistically significant with each clinical stage; estimate scores were negatively correlated with stage (*p* = 0.04) and stage T (*p* = 0.04) (Figures 6B,C). In contrast, in the CCL17 low-expression group, the immune correlation score did not significantly correlate with each clinical stage (*p* > 0.05) (Supplementary Figures S3B-C). It is suggested that the immune and stromal components in the presence of CCL17 high expression are associated with the clinicopathological characteristics of LUAD patients.

### Comparison of the Correlation Between CCL17 High- and Low-Expression Groups and 22 TIIC Subtypes Using the CIBERSORT Algorithm

**FIGURE 7:**
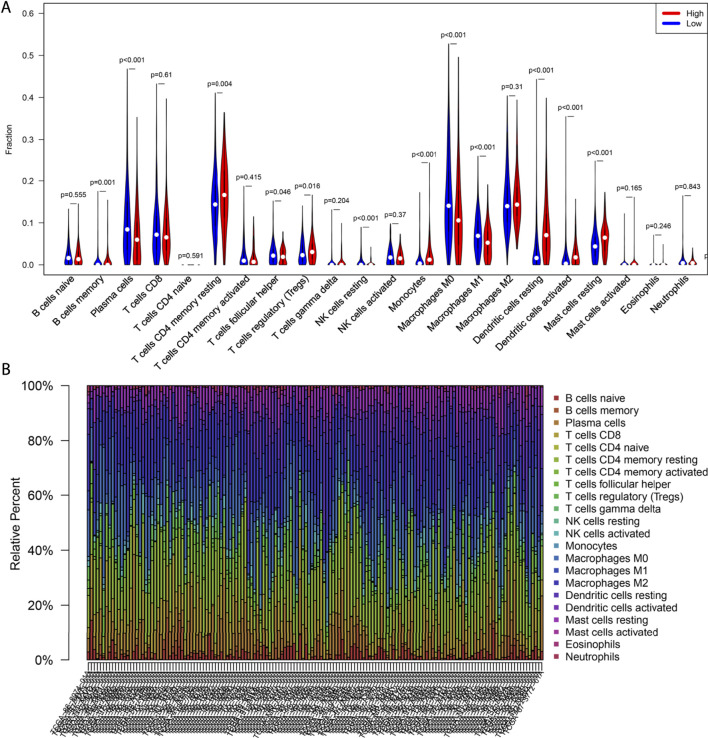
Correlation between CCL17 expression and immune cell infiltration. **(A)** Violin plot of CCL17 high- and low-expression groups in LUAD patients. **(B)** Proportion of 22 immune cells in LUAD in the CCL17 high-expression group.

The CIBERSORT method was used to estimate the immune cell composition of 491 LUAD samples and quantify the relative levels of different cell types in a mixed cell population. In terms of immune infiltration in the TCGA-LUAD cohort, there was a significant difference between the CCL17 high- and low-expression groups. As shown in Figure 7A, there were 12 types of immune cells affected by CCL17 expression. Among them, the proportions of B cells memory (*p* = 0.001), T cells CD4 memory resting (*p* < 0.001), monocytes (*p* < 0.001), dendritic cells resting (*p* < 0.001), dendritic cells activated (*p* < 0.001), mast cells resting (*p* < 0.001), and Treg cells (*p* = 0.016) in the CCL17 high-expression group were significantly higher than those of the CCL17 low-expression group. Subsequently, further by bar graph (Figure 7B), the components of 22 TIIL subtypes in the CCL17 high-expression group were clearly shown, and the main components were T cells CD4 memory resting, dendritic cells resting, T cells CD8, plasma cells, mast cells, dendritic cells activated, and monocytes and mast cells resting. The aforementioned results further revealed the role of CCL17 expression in regulating tumor immunology in LUAD.

### Correlation of 22 Immune Cells With OS and Clinical Stage of LUAD

**FIGURE 8:**
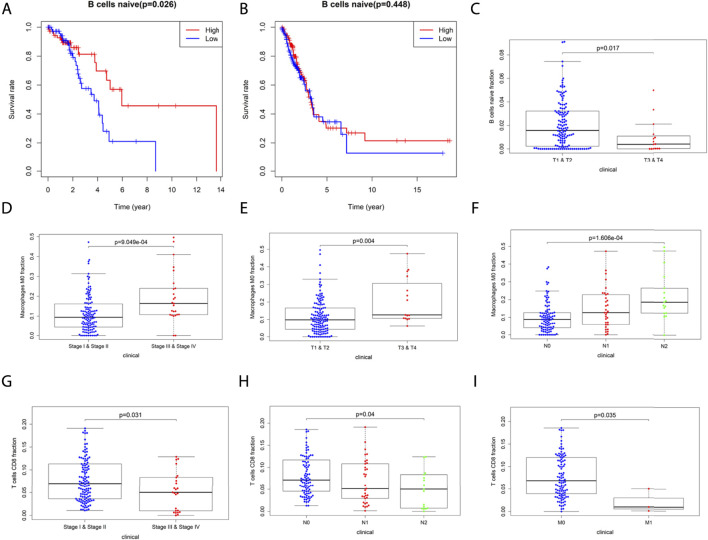
**(A-B)** Correlation of naive B cells with overall survival in LUAD patients in the CCL17 high- **(A)** and low- **(B)** expression groups. **(C-I)** Correlation of 22 immune cells with the clinical stages (stage, TNM stage) of LUAD patients in the CCL17 high-expression group. **(C)** Correlation of naive B cells infiltration with clinical stages (stage T) of LUAD patients. **(D-F)** Correlation of M0 macrophage infiltration with clinical stages (stage, stage T, and N) of LUAD patients. **(G-I)** Correlation of T cells CD8 infiltration with clinical stages (stage, stage N, and M) of LUAD patients.

We further analyzed the association between OS and TIICs abundance in the TCGA-LUAD cohort and found that in the CCL17 high-expression group, high expression of naive B cells had a positive prognostic effect on LUAD patients (OS, *p* = 0.026) (Figure 8A). However, naive B cells had no effect on the overall survival of LUAD patients in the CCL17 low-expression group (OS, *p* > 0.05) (Figure 8B).

The infiltration of 22 TIIL subtypes in LUAD patients at different clinical stages (stage, TNM stage) caused by differences in CCL17 expression levels was also investigated. The results showed that in the CCL17 high-expression group, there was a significant correlation between the expression of certain immune cells and each stage, while in the CCL17 low-expression group, there was no correlation between these immune cells and the clinical stages.

As shown in Figure 8C, the infiltration abundance of naive B cells was significantly correlated with the stage T in the CCL17 high-expression group (*p* = 0.017). The abundance of M0 macrophage infiltration was significantly correlated with stage (*p* < 0.001) and stage T (*p* = 0.004) and stage N (*p* < 0.001), and all were positively correlated (Figures 8D–F). The infiltration abundance of CD8^+^ T cells was negatively correlated with stage (*p* = 0.031) and stage N (*p* = 0.04) and stage M (*p* = 0.035) (Figures 8G–I). The aforementioned results showed that patients with early LUAD (stages I and II) exhibited more naive B cells and CD8^+^ T cells, while patients with late LUAD (stages III and IV) had a higher content of M0 macrophages (Figures 8C–I).

### Mutation Profile of CCL17 in LUAD

**FIGURE 9:**
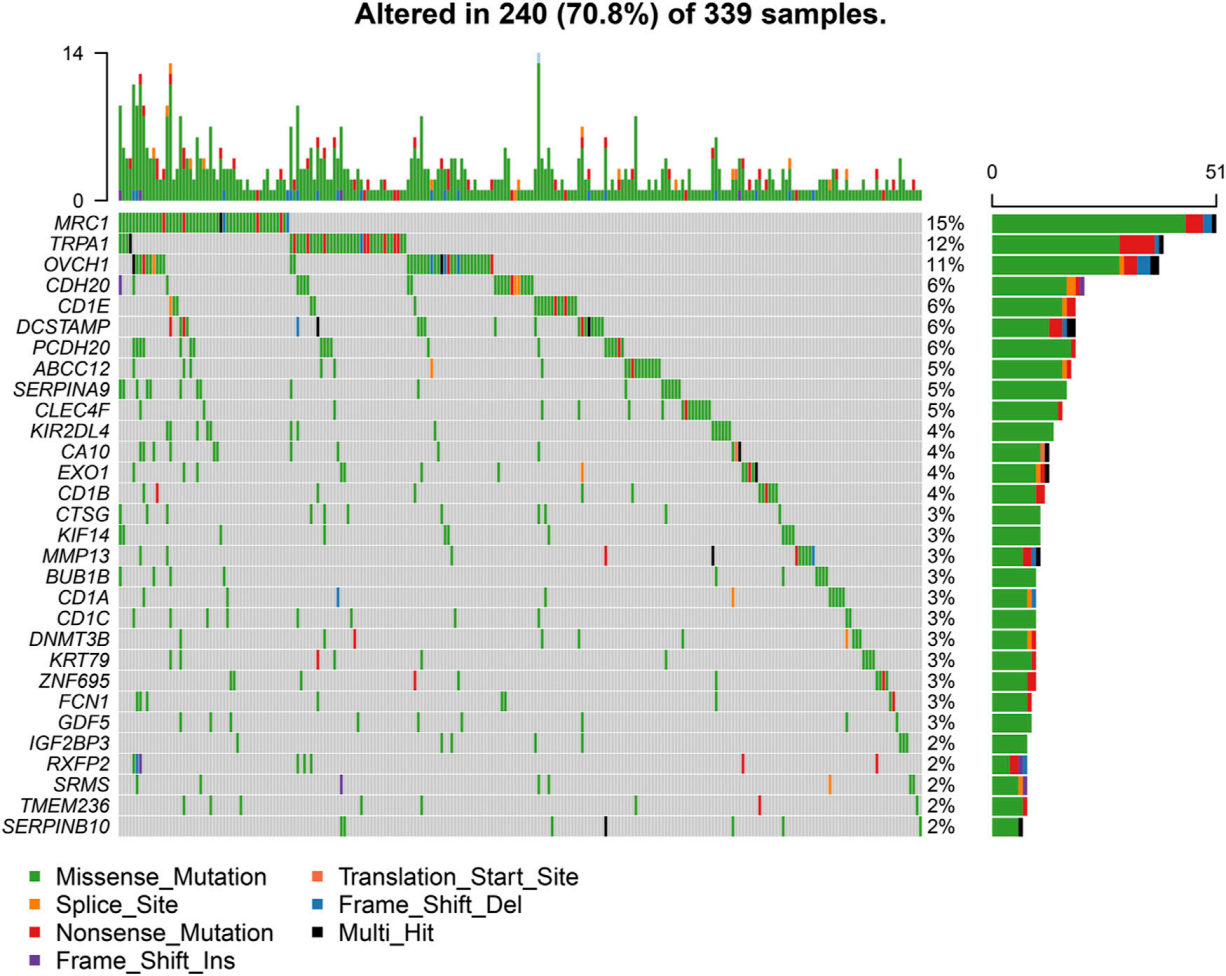
Waterfall plot of mutated genes in the CCL17-related module genes in TCGA-LUAD. The different color annotations in the waterfall plot represent different mutation types, and the plot shows the mutation frequencies of the top 30 genes in the sample.

**FIGURE 10:**
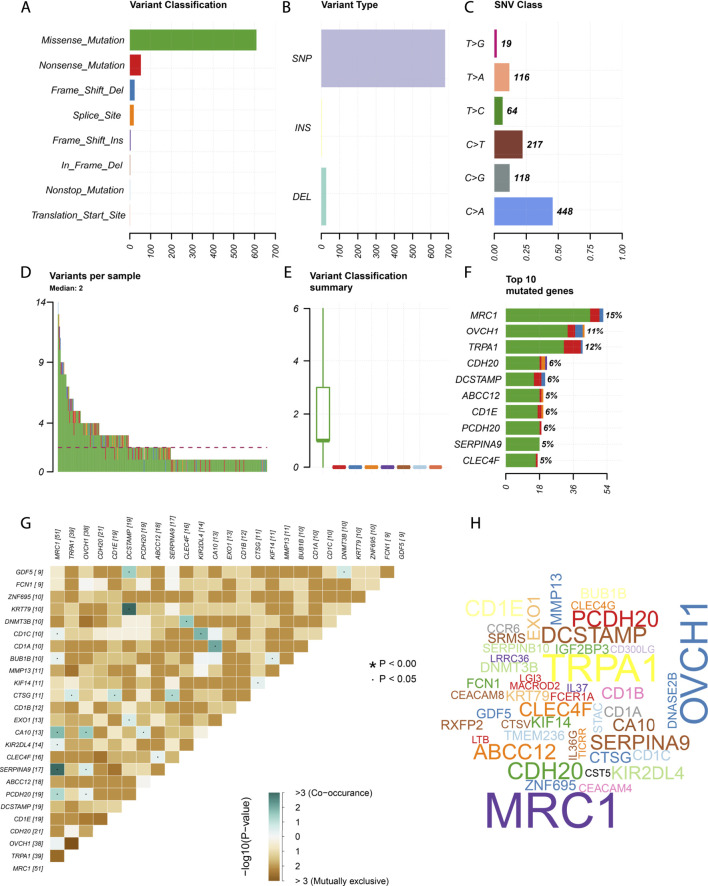
Summary of mutation information in the CCL17-related module genes in TCGA-LUAD samples. **(A-C)** Variant classification **(A)**, variant type **(B)**, and SNV class **(C)**. **(D-E)** variants in per sample and summary of variant classification in each sample. **(F)** Top 10 genes mutated. **(G)** Co-expression analysis between mutant genes. **(H)** Genecloud plots show the mutation frequencies of most genes, with larger fonts representing higher mutation frequencies.

We downloaded somatic mutation data of LUAD patients from the TCGA database and used the “maftools” package to visualize the results of the mutation data. We present the mutation information of the blue module genes significantly associated with CCL17 in the WGCNA analysis and all genes in TCGA-LUAD in the form of waterfall plots (Figure 9 and Supplementary Figure S4). We found that the frequency of mutations in CCL17-related module genes was lower in LUAD patients. In addition, we further classified these mutations in CCL17-related module genes according to different classification categories, where missense mutations were predominant (Figure 10A), single nucleotide polymorphisms occurred more frequently than insertions or deletions (Figure 10B), and C > A was the most common single nucleotide variant (SNV) in LUAD (Figure 10C). In addition, we also counted the number of altered bases in each sample and showed the mutation types in different colors in the box plot of LUAD (Figure 10D,E). Finally, we showed the top 10 mutated genes in CCL17-related module, including *MRC1* (15%), *TRPA1* (12%), and other genes (Figure 10F), and found that CCL17 was not a high mutation frequency gene in LUAD. Figure 10G show the concordance and mutually exclusive associations among mutant genes, where blue indicates co-occurrence and brown indicates mutually exclusive relationships. The mutation frequencies of most genes are shown by the Genecloud plot in Figure 10H.

**FIGURE 11:**
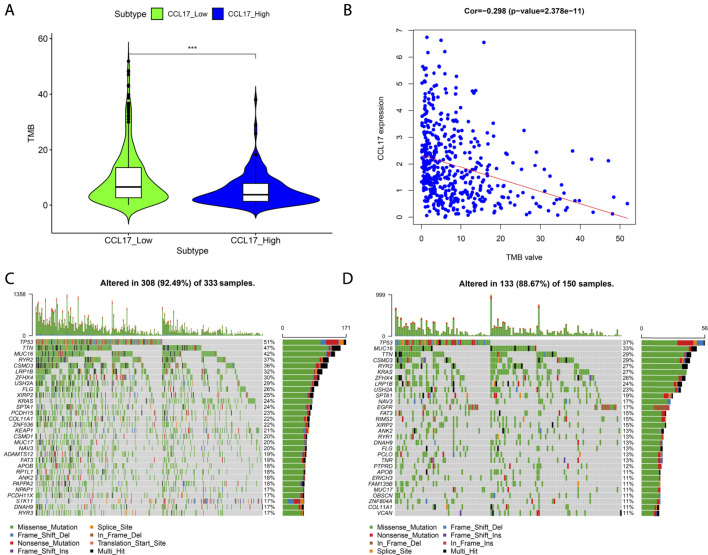
**(A)** Box plot of the difference in TMB values between CCL17 high- and low-expression groups. **(B)** Correlation between CCL17 expression and TMB values. **(C-D)** Waterfall plots of mutated genes in the CCL17 low- (C) and high-expression (D) groups.

TMB values and the differences in TMB values according to the grouping of CCL17 high- and low-expression levels were calculated for each sample. We found that the TMB values in the CCL17 high-expression group were significantly lower than those in the CCL17 low-expression group (*p* < 0.001) (Figure 11A), and the expression levels of CCL17 and TMB values were negatively correlated (Figure 11B). It indicates that the somatic cells in the CCL17 low-expression group had a higher degree of mutation and a higher tumor burden. In addition, waterfall plots of mutated genes in the CCL17 high- and low-expression groups showed that the mutation frequency of genes in the CCL17 low-expression group (such as *TP53*, *MUC16*, and *TTN*) was generally higher than that in the CCL17 high-expression group (Figures 11C,D). The mutation frequency of *TP53* in CCL17 high- and low-expression groups is 37 and 51%, respectively.

**FIGURE 12:**
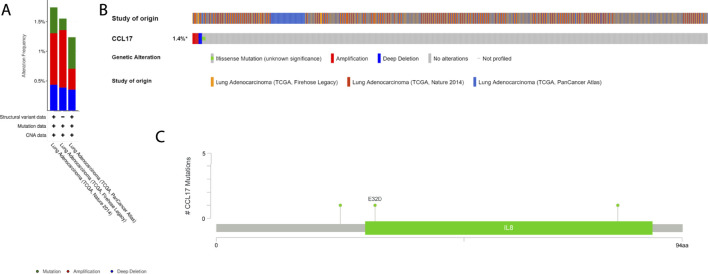
Mutation status view of CCL17 in LUAD. **(A)** Histogram of the alteration frequencies of CCL17 in LUAD studies. **(B)** OncoPrint tab summarizes genomic alterations in CCL17 across a sample set. Each column represents a tumor sample. Red bars indicate gene amplifications, blue bars are deep deletions, and green squares are missense mutation. **(C)** Graphical view shows the Pfam protein structural domain and specific mutation sites of CCL17.

Next, we obtained the histogram of the alteration frequency of CCL17 in the TCGA-LUAD dataset through the cBioPortal database. The results showed that the mutation frequency of CCL17 in LUAD was only about 1.4% (Figure 12A,B). To further understand the CCL17 mutations, we show the Pfam protein structural domain of CCL17 and the position of specific mutations by graphical view. The length of the line connecting the mutation annotation to the protein indicates the number of samples with mutations. The most recurrent mutations are marked in the graphical view (Figure 12C). The results show that all five mutations in CCL17 are missense mutations in LUAD, while we provide additional information on all mutations in CCL17 through the tabular view (Supplementary Table S1).

### Clinical Validation of CCL17 and Immune Cell Marker Molecules in Terms of Protein Expression

**FIGURE 13:**
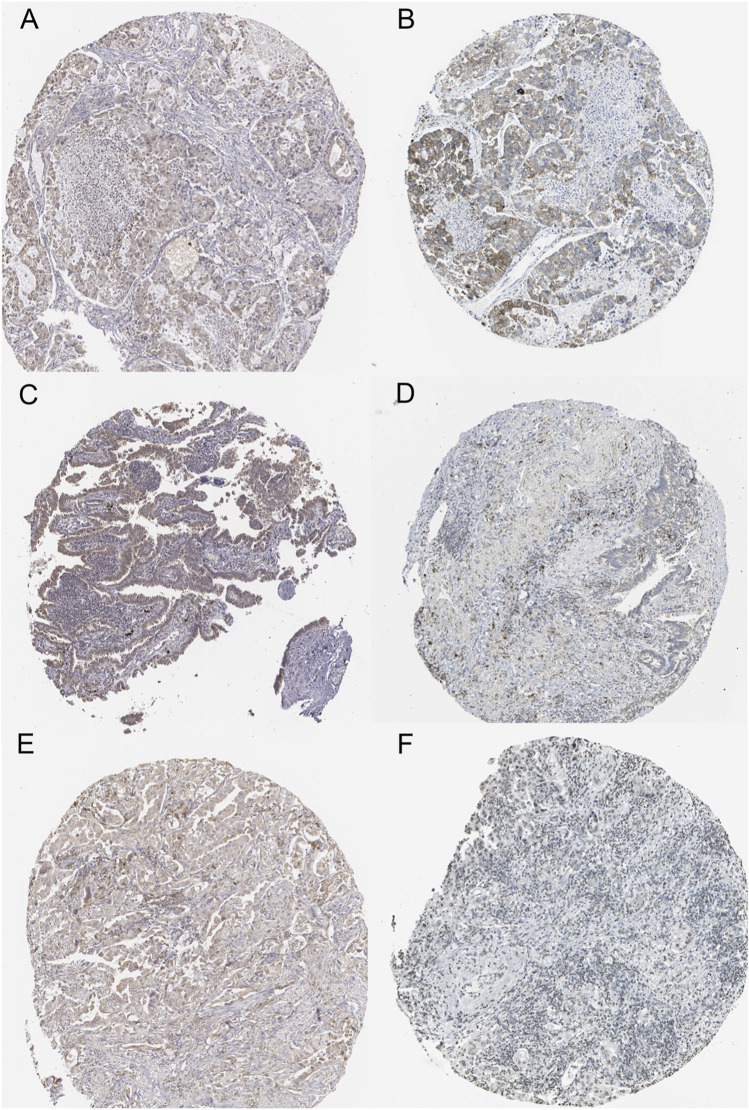
Immunohistochemical staining images of LUAD tumor tissues of CCL17 and specific immune cell marker molecules in the HPA database. **(A-B)** Immunohistochemical staining images of CCL17 **(A)** and CD3G **(B)** in LUAD patient number 2041. **(C-D)** Immunohistochemical staining images of CCL17 **(C)** and CD4 **(D)** in LUAD patient number 2438. **(E-F)** Immunohistochemical staining images of CCL17**(E)** and CD79B **(F)** in LUAD patient number 2403.

To validate the protein expression of CCL17 and CCL17-affected immune cells in LUAD patients, we collected and examined immunohistochemical staining images and clinical information of tumor tissues from the HPA database of three LUAD patients. Immunohistochemical staining of the tumor tissue of one 51-year-old female LUAD patient showed some degree of protein expression of both CCL17 and CD3G (CD4/CD8^+^ T cell marker molecule). Patient number: 2041; CCL17 immunohistochemical staining status: low; intensity: weak; quantity: >75%; CD3G immunohistochemical staining status: medium; intensity: moderate; quantity: >75% (Figure 13A,B). Immunohistochemical staining of the tumor tissue of one 64-year-old female LUAD patient showed some degree of protein expression of both CCL17 and CD4 (CD4^+^ T cell marker molecule). Patient number: 2438; CCL17 immunohistochemical staining: low; intensity: weak; quantity: >75%; CD4 immunohistochemical staining status: low; intensity: weak; quantity: >75% (Figure 13C,D). Meanwhile, immunohistochemical staining of the tumor tissue of another 65-year-old female patient with LUAD showed some degree of protein expression of both CCL17 and CD79B (naive B cells marker molecule). Patient number: 2403; CCL17 immunohistochemical staining: low; intensity: weak; quantity: >75%; CD79B immunohistochemical staining: low; intensity: weak; quantity: 75–25% (Figure 13E,F).

### Flow Cytometry Assay

**FIGURE14:**
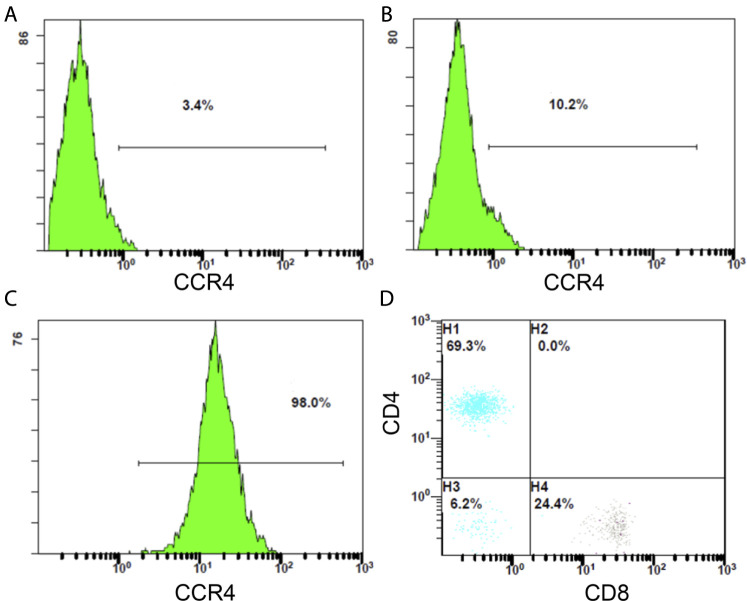
**(A)** Proportion of CCR4-positive lymphocytes in PBMC before tumor antigen stimulation. **(B)** Proportion of CCR4-positive lymphocytes in PBMC after tumor antigen stimulation. **(C)** Flow sorting purification results of CCR4-positive lymphocytes. **(D)** Proportion of CD4-positive and CD8-positive cells in CCR4-positive lymphocytes.

We examined PBMCs before and after tumor antigen stimulation, and as shown in Figures 14A,B, the percentage of CCR4-positive lymphocytes increased after induction. After sorting out the CCR4-positive lymphocytes using a MoFlo cytometer (Figure 14C), we further detected the proportion of CD4^+^ and CD8^+^ cells among the pairs of CCR4+ cells by flow cytometry. The results are shown in Figure 14D, and CCR4+CD4^+^ cells accounted for 69.3%, and CCR4+CD8^+^ cells accounted for 24.4%.

### Transwell Chemotaxis and Cytotoxicity Assay

**FIGURE 15:**
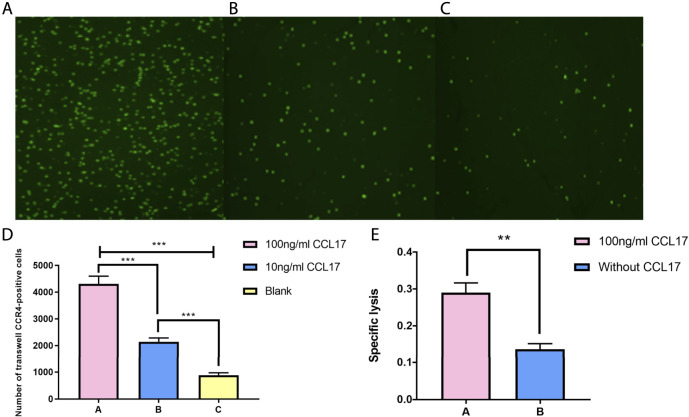
**(A-C)** CFSE staining of CCR4-positive lymphocytes in the lower chamber under different concentrations of CCL17 chemotaxis. **(A)** 100 ng/ml CCL17, **(B)** 10 ng/ml CCL17, and **(C)** blank. **(D)** Number of CCR4-positive lymphocytes chemotactic to the lower chamber at different concentrations of CCL17 (***p < 0.001). **(E)** Specific lysis of CCR4-positive lymphocytes in the lower chamber on LUAD H1993 cells (*p < 0.05).

To verify the effect of CCL17 on local immune cell infiltration in LUAD cell tumors, we constructed a transwell-based cell infiltration assay model. As shown in Figures 15A–C, the chemotactic effect of the treatment group (lower chamber with CCL17) on CCR4-positive cells was significantly higher than that of the control group (lower chamber without CCL17). Also, within a certain concentration range, the higher the concentration of chemokine CCL17 had a stronger chemotactic ability on CCR4-positive cells (*p* < 0.001) (Figure 15D). To further verify the effect of CCL17-induced immune cells on lung adenocarcinoma cell function, we examined the cytotoxic effect of immune cells passing through transwell plates. As shown in Figure 15E, after induction of CCR4-positive lymphocytes into the lower chamber, the specific lysis index of treatment group A and treatment group B increased to 0.29 ± 0.026 and 0.137 ± 0.015, respectively. The specific lysis of CCR4-positive cells in the lower chamber of treatment group A (100 ng/ml CCL17) on HLA-A2-positive lung adenocarcinoma H1993 cells was significantly stronger than that of treatment group B (without CCL17), and the difference was significant (*p* < 0.05). It indicates that CCL17 contributes to the chemotactic effect of CCR4-positive lymphocytes to the TME, which can enhance the specific lysis on LUAD H1993 cells.

## Discussion

Although the incidence of lung cancer has declined in recent years, it still ranks first in the world in terms of mortality. LUSC and LUAD are the main pathological types of NSCLC, which accounts for 85% of all lung cancers. Compared to patients with LUSC, patients with LUAD have a poor prognosis, accounting for nearly 40% of NSCLC-related mortality (Shen et al., 2019). Chemokine receptors are widely expressed on cells in the TME, including tumor cells. Chemokines affect a variety of processes such as leukocyte recruitment, tumor cell survival, tumor cell adhesion, proliferation, immune suppression, invasion, and metastasis, and thus the role of chemokines in tumor immunity has received increasing attention (Borsig et al., 2014; Kang et al., 2019). Some controversy exists about whether chemokine CCL17 exerts antitumor effects or suppresses immune responses in the NSCLC microenvironment (Nakanishi et al., 2006; de Chaisemartin et al., 2011). For these reasons, we seek to clarify the biological significance and prognostic value of CCL17 in NSCLC.

In this study, we found that the expression levels of CCL17 were significantly different from normal tissues in both LUAD and LUSC in TIMER database analysis. Subsequently, the Kaplan–Meier plotter database was used to analyze the OS and PFS of CCL17 in lung cancer patients at various clinical stages. We found that high expression of CCL17 had a good prognostic effect on lung cancer patients in the early stage (stages I–II), while it was not significant for patients with advanced lung cancer (stages III–IV). Notably, the significant positive prognostic effect of CCL17 held true only for adenocarcinoma tissue among lung cancer types but not for squamous cell carcinoma.

The correlation of CCL17 with immune cell infiltration and immune molecular features in the TME of LUAD was analyzed using TISIDB databases. The results showed that CCL17 expression levels were positively correlated with TILs, such as (effector memory T cell) tem_CD8 cell and macrophages. CCL17 was also found to be positively correlated with immunostimulators such as CD28 and CD80, which are associated with B cell and T cell activation. We also observed a positive correlation with MHCs located in specialized presenting cells such as dendritic cells and macrophages and involvement in antigen presentation as well as T cell differentiation, such as HLA-DPA1, HLA-DPB1, and HLA-DRA. Dendritic cells are the most functional and specialized antigen-presenting cells in the body, and they efficiently uptake, process, and present antigens. Immature dendritic cells have strong migration ability, whereas mature dendritic cells can effectively activate the T cells naive and are at the center of initiating, regulating, and maintaining the immune response (Kumai et al., 2015; Altman et al., 2018).

We then downloaded the gene expression profile matrix of 491 TCGA-LUAD patients from the UCSC Xena database and performed differential expression gene analysis based on CCL17 high-and low-expression groupings and screened 1,455 differential genes. WGCNA analysis with these genes revealed the highest negative correlation between the blue module, where the *CCL17* gene is located, and LUAD clinical stages and survival status. This correlation suggests that low expression of CCL17 is detrimental to the survival of patients. In addition, the *CCR4* receptor gene is also one of the key genes in the blue module where CCL17 is located. Next, GO and KEGG enrichment analysis of the genes in the blue module revealed important biological processes including molecular functions such as “leukocyte migration,” “cell chemotaxis,” “lymphocyte migration,” and “antigen processing and presentation.” KEGG pathway analysis showed that the blue module gene plays a key role in the cytokine–cytokine receptor interaction pathway, chemokine signaling pathway, and cancer pathway. This indicates that the blue module gene mainly acts on the immune-chemotactic reaction in the TME of LUAD, which improves the prognosis of LUAD patients.

Using ESTIMATE algorithm of the R package, it was found that the immune and stromal scores of the CCL17 high-expression group were significantly higher than those of CCL17 low-expression group. The mean immune score of the former (1,917.50) was significantly higher than that of the latter (1,278.45), implying that the CCL17 high-expression group was more immune than the CCL17 low-expression group in LUAD patients. We found that in the CCL17 high-expression group, the higher immune score had a positive effect on the OS of LUAD patients. The CCL17 high-expression group had a higher immune score in stages I and II and stages T1 and T2 and a negative correlation between the immune score and clinical stage (stage and stage T) (*p* < 0.05).

We further analyzed the distribution of 22 TIIL subtypes in TCGA-LUAD samples by the CIBERSORT algorithm. We found that the proportions of B cells memory, T cells CD4 memory resting, monocytes, dendritic cells resting, dendritic cells activated, mast cells resting, and Treg cells in the CCL17 high-expression group were significantly higher than those in the CCL17 low-expression group. T cells CD4 memory resting displayed the highest degree of infiltration in the CCL17 high-expression group. T cells can be divided into many types according to their function and surface markers, and CD4^+^ memory T cells represent one of the types (Castellino et al., 2006). These cells maintain immune memory and play an immunoprotective role during tumor metastasis, serve in an intermediate step in the immune response, and can proliferate and spread to activate other types of immune cells that generate direct immune responses (Soon et al., 2019).

High expression of naive B cells in the CCL17 high-expression group had a positive prognostic effect on LUAD patients (OS, *p* = 0.026). High expression of CCL17 affected the distribution of three important immune cells in early and late stages of LUAD patients, namely increased CD8^+^ T cell and naive B cells infiltration and decreased M0 macrophages infiltration in early stages and decreased CD8^+^ T cell and naive B cells infiltration and increased M0 macrophages infiltration in late stages. These findings indicate that high expression of CCL17 may alter the immune response status and thus affect the tumor progression. Macrophages are commonly abundant cells in the TME and are plastic and heterogenous immune cells. The primary phenotype of a macrophage without stimulation or in the resting cells is referred to as an M0 macrophage. M0 macrophages can be induced to M1 or M2 polarization by environmental signals (Martinez, 2011; Miao et al., 2017). The polarization of macrophages is crucial for their function and plays different roles in immune regulation, inflammation, tissue remodeling, proliferation, and metabolism (Miao et al., 2017). M1 macrophages enhance antitumor immunity, whereas M2 macrophages are associated with immunosuppression and tumor progression (Schultze et al., 2015). The human peripheral blood B cell population is composed of multiple subpopulations, and most of them are naive B cells. “Naive B cells” refer to B cells that are not stimulated by antigens. We know that to produce antibodies and exert a stronger immune function, B cells must be activated first (Rowland et al., 2012). B cells are important immune cells in the immune system, and their main function is to mediate fluid immunity. B cells are also important antigen-presenting cells that can ingest, process, and present antigens (Hua and Hou, 2020). CD8^+^ T cells are important effectors of cell-mediated immunity (Ma et al., 2016). Cytotoxic T lymphocytes and CD8 surface proteins are called CD8^+^ T cells (Wang et al., 2017). CD8^+^ T cells recognize the antigen peptide-MHC-I complex and costimulatory molecules on the surface of tumor cells through T cell receptor (TCR) to initiate the killing process (Henning et al., 2018). The interaction between CD4^+^ T follicular helper and B cells plays an important role in the induction of antitumor immune responses and may enhance the effector function of tumor-infiltrating CD8^+^ T cells in lung cancer (Cui et al., 2020).

To understand the mutation profile of CCL17 in LUAD, we used the “maftools” package to visualize somatic mutation data from TCGA-LUAD patients. We found a low frequency of mutations in module genes associated with CCL17 in LUAD patients. We also proved that LUAD patients with low expression of CCL17 had a higher rate of TP53 mutations. TP53 mutations occur frequently in cancer and are associated with poor prognosis in a variety of cancers (Dong et al., 2017). In addition, we calculated the TMB value for each sample, which is the number of nonsynonymous mutations in somatic cells within a specific genomic region, usually expressed as the number of mutations per megabase (Hellmann et al., 2018; Klebanov et al., 2019). The TMB value is the average number of mutations in the tumor genome, including the total number of gene-coding errors, base substitutions, and gene insertion or deletion errors. In this study, we found that the TMB values in the CCL17 low-expression group were significantly higher than those in the CCL17 high-expression group (*p* < 0.001), suggesting that the degree of somatic mutation is higher and the increased tumor burden on the organism may be relatively more pronounced in the CCL17 low-expression group. Therefore, the poor prognosis for patients in the CCL17 low-expression group may be related to their higher TMB values and decreased autoimmune infiltration of the organism.

We validated the protein expression of CCL17 and CCL17-affected immune cells in LUAD patients using the HPA database. We collected and examined immunohistochemical staining images and clinical information about tumor tissues from three LUAD patients. The results showed that CCL17 and CD3G (CD4/CD8^+^ T cell marker molecule), CD4 (CD4^+^ T cell marker molecule), and CD79B (naive B-cell marker molecule) were expressed to some extent in tumor tissues of three LUAD patients without dysregulation.

In addition, previous studies have shown that CCL17 is an important chemokine that exerts chemotactic effects on CCR4+ T lymphocytes via the receptor molecule CCR4 and may play a vital immunomodulatory role in the TME (Viola et al., 2012; Yoshie and Matsushima, 2015).Thus, we confirmed by transwell cell chemotaxis assay that the higher the concentration of CCL17, the stronger the chemotaxis to CCR4-positive lymphocytes. The cytotoxicity assay also showed that CCL17 contributed to the migration of CCR4-positive lymphocytes into the LUAD H1993 TME and enhanced the specific lysis of the LUAD cells.

In our study, we found that the mRNA expression level of CCL17 was negatively correlated with the clinical stages (stage, TNM stage) of LUAD patients. The OS of patients with high expression of CCL17 was higher than that of patients with the CCL17 low-expression level, which had a positive effect on the prognosis of patients with early LUAD in particular. By further analyzing the effect of CCL17 high- and low-expression level on immune cell infiltration in the TME of TCGA-LUAD, we found that CCL17 may influence the level of immune cell infiltration in each clinical stage of LUAD patients, with a higher distribution of macrophages M0 in late-stages (stages III and IV) and a higher distribution of naive B cells and CD8^+^ T cells in early-stages (stages I and II). Furthermore, we found that the TMB values in the CCL17 low-expression group were significantly higher than those in the CCL17 high-expression group. The transwell chemotaxis assay and cytotoxicity assay confirmed that CCL17 contributed to the chemotactic effect of CCR4-positive lymphocytes to the H1993 LUAD TME and enhanced the specific lysis of LUAD cells.

## Conclusion

In conclusion, we used different bioinformatics methods and *in vitro* experiments to explore and validate the biological significance of CCL17 in LUAD sequencing data from public databases. This study clarifies that high expression of CCL17 in early LUAD patients promoted local immune cell infiltration. We believe that these findings provide a useful reference for understanding the intrinsic relationship and molecular mechanisms of CCL17 in LUAD and may eventually have an impact in the clinical setting.

## Data Availability

The original contributions presented in the study are included in the article/Supplementary Material, further inquiries can be directed to the corresponding author.

## Abbreviations

LUAD, lung adenocarcinoma; LUSC, lung squamous cell carcinoma; MHCs, major histocompatibility complexes; NSCLC, non-small cell lung cancer; OS, overall survival; PFS, progression-free survival; TME, tumor microenvironment; TMB, tumor mutation burden; TILs, tumor-infiltrating lymphocytes; WGCNA, weighted correlation network analysis.
