# Supplementary material for: Chemokine CCL17 Affects Local Immune Infiltration Characteristics and Early Prognosis Value of Lung Adenocarcinoma
Source: Front Cell Dev Biol. 2022 Mar 7;10:816927. doi: 10.3389/fcell.2022.816927 (PMC8936957; doi:10.3389/fcell.2022.816927)
Supplement: Supplementary file 1 [file DataSheet1.zip › Supplementary data/SupplementaryFigure Legends.docx]

**Supplementary Figure Legends**

**Supplementary Figure S1.** Venn diagram of TCGA-LUAD differential genes by DESeq2 and edgeR analysis.

**Supplementary Figure S2.** The figure show the scale-free fit index and the mean connectivity for various soft-thresholding powers, respectively. **(A)**The vertical axis is R^2^ (the evaluation parameter of the scale-free network), the horizontal line is drawn at 0.9. The soft threshold of 4 is the first breakthrough of R^2^, reaching 0.9, when the network is already in line with the non-scale distribution. **(B)**When the soft-thresholding powers (β) equaled four, the average degree of connectivity was close to zero.

**Supplementary Figure S3**. **(A)**Correlation between survival and score in LUAD patients in the CCL17 low-expression group. **(B)**Stage classification of CCL17 low-expression group. **(C)**T classification of CCL17 low-expression group.

**Supplementary Figure S4.** Waterfall plots for each mutated gene in each TCGA-LUAD.

**Supplementary Table1.** This table provides information on the mutation status of CCL17 in patients with LUAD.
