# Supplementary material for: Chemokine CCL17 Affects Local Immune Infiltration Characteristics and Early Prognosis Value of Lung Adenocarcinoma
Source: Front Cell Dev Biol. 2022 Mar 7;10:816927. doi: 10.3389/fcell.2022.816927 (PMC8936957; doi:10.3389/fcell.2022.816927)
Supplement: Supplementary file 1 [file DataSheet1.zip › Supplementary data/Table1.docx]

**Table 1. Correlation between CCL17 expression level and prognosis in lung cancer with different clinicopathological features by Kaplan-Meier plotter.**

| **Clinicopathological Overall survival Progression-free survival**  **characteristics (n = 1927) (n=982)** | | | | | | |
| --- | --- | --- | --- | --- | --- | --- |
|  | **N Hazard ratio P-value** | | | **N Hazard ratio P-value** | | |
| **SEX** | | | | | | |
| Female | 714 | **0.74(0.58-0.93)** | **0.01** | 468 | **0.65(0.49-0.87)** | **0.0032** |
| Male | 1100 | **0.74(0.63-0.87)** | **0.00018** | 514 | **0.72(0.56-0.93)** | **0.012** |
| **STAGE** | | | | | | |
| 1 | 577 | **0.59(0.45-0.78)** | **0.00018** | 325 | **0.55(0.36-0.85)** | **0.0064** |
| 2 | 244 | 0.85(0.58-1.25) | 0.41 | 130 | 1.43(2.84-2.44) | 0.1851 |
| 3 | 70 | 0.6(0.33-1.1) | 0.094 |  |  |  |
| **STAGE T** | | | | | | |
| 1 | 437 | **0.63(0.46-0.86)** | **0.0029** | 172 | **0.39(0.24-0.65)** | **0.0002** |
| 2 | 589 | 0.83(0.65-1.06) | 0.13 | 351 | 0.78(0.55-1.1) | 0.1476 |
| 3 | 81 | **2.15(1.26-3.66)** | **0.0042** | 21 | 0.56(0.21-1.53) | 0.2551 |
| 4 | 46 | 0.63(0.34-1.18) | 0.15 |  |  |  |
| STAGE N | | | | | | |
| 0 | 781 | **0.73(0.53-0.93)** | **0.012** | 374 | **0.7(0.49-0.98)** | **0.0366** |
| 1 | 252 | **0.72(0.5-0.99)** | **0.0445** | 130 | 0.72(0.34-1.2) | 0.2026 |
| 2 | 111 | 0.82(0.52-1.28) | 0.37 | 51 | 1.35(0.65-2.82) | 0.4235 |
| **STAGE M** | | | | | | |
| 0 | 681 | **0.68(0.54-0.87)** | **0.0015** | 195 | 0.73(0.43-1.23) | 0.2321 |
| **Histology** | | | | | | |
| adenocarcinoma | 719 | **0.68(0.54-0.86)** | **0.0011** | 461 | **0.59(0.43-0.82)** | **0.0012** |
| squamous cell carcinoma | 524 | 0.86(0.68-1.1) | 0.23 | 141 | 1.26(0.72-2.19) | 0.4209 |
| **Smoking history** | | | | | | |
| exclude those never smoked | 820 | **0.79(0.63-0.99)** | **0.037** | 603 | **0.6(0.39-0.94)** | **0.0238** |
| only those never smoked | 205 | **1.76(1-3.11)** | **0.0491** | 193 | **0.55(0.34-0.89)** | **0.0144** |
