# Supplementary figures and images for: Chemokine CCL17 Affects Local Immune Infiltration Characteristics and Early Prognosis Value of Lung Adenocarcinoma
Source: Front Cell Dev Biol. 2022 Mar 7;10:816927. doi: 10.3389/fcell.2022.816927 (PMC8936957; doi:10.3389/fcell.2022.816927)

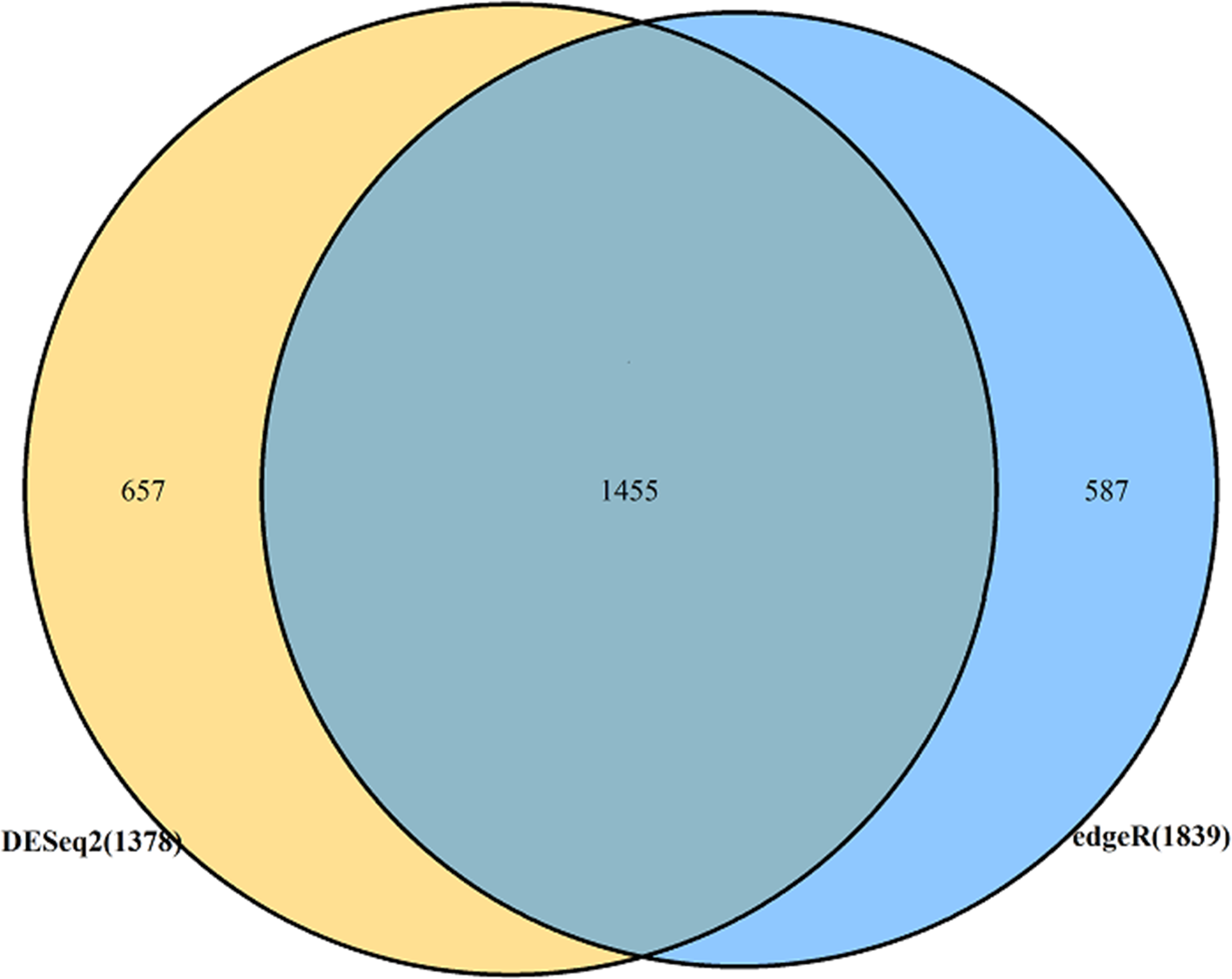

Supplement: Supplementary file 1 [file DataSheet1.zip › Supplementary data/Supplementary Figure S1.tif]

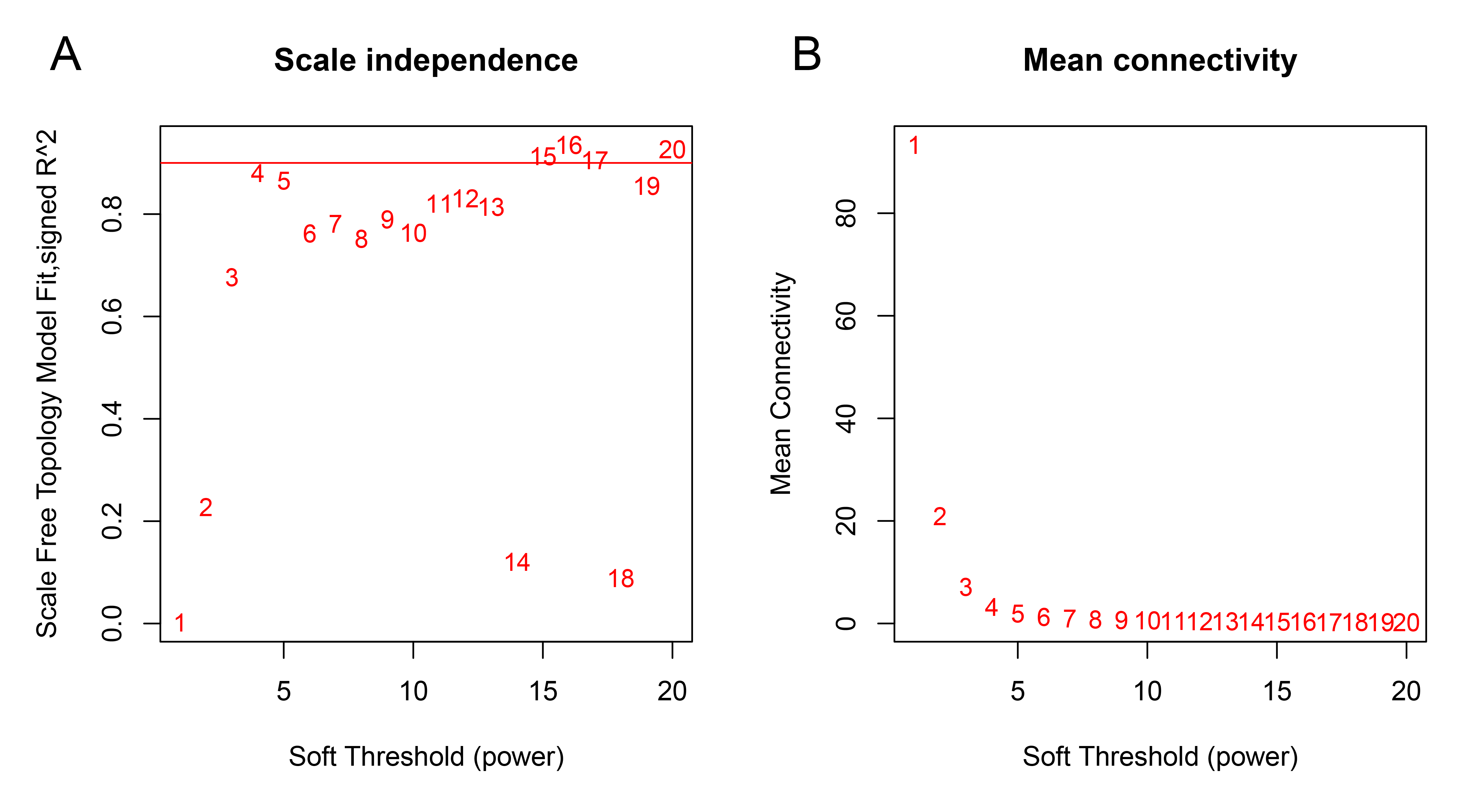

Supplement: Supplementary file 1 [file DataSheet1.zip › Supplementary data/Supplementary Figure S2.tif]

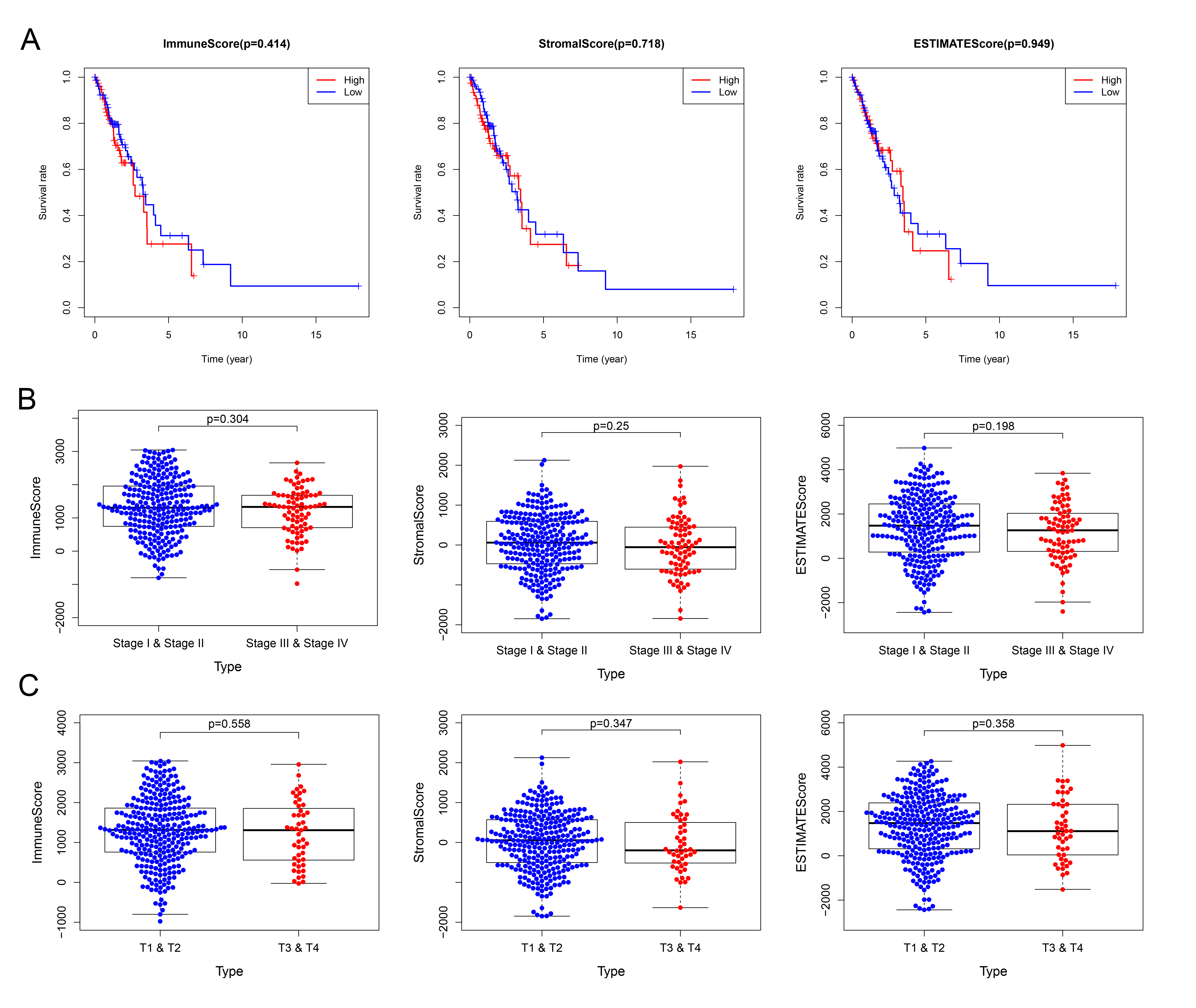

Supplement: Supplementary file 1 [file DataSheet1.zip › Supplementary data/Supplementary Figure S3.tif]

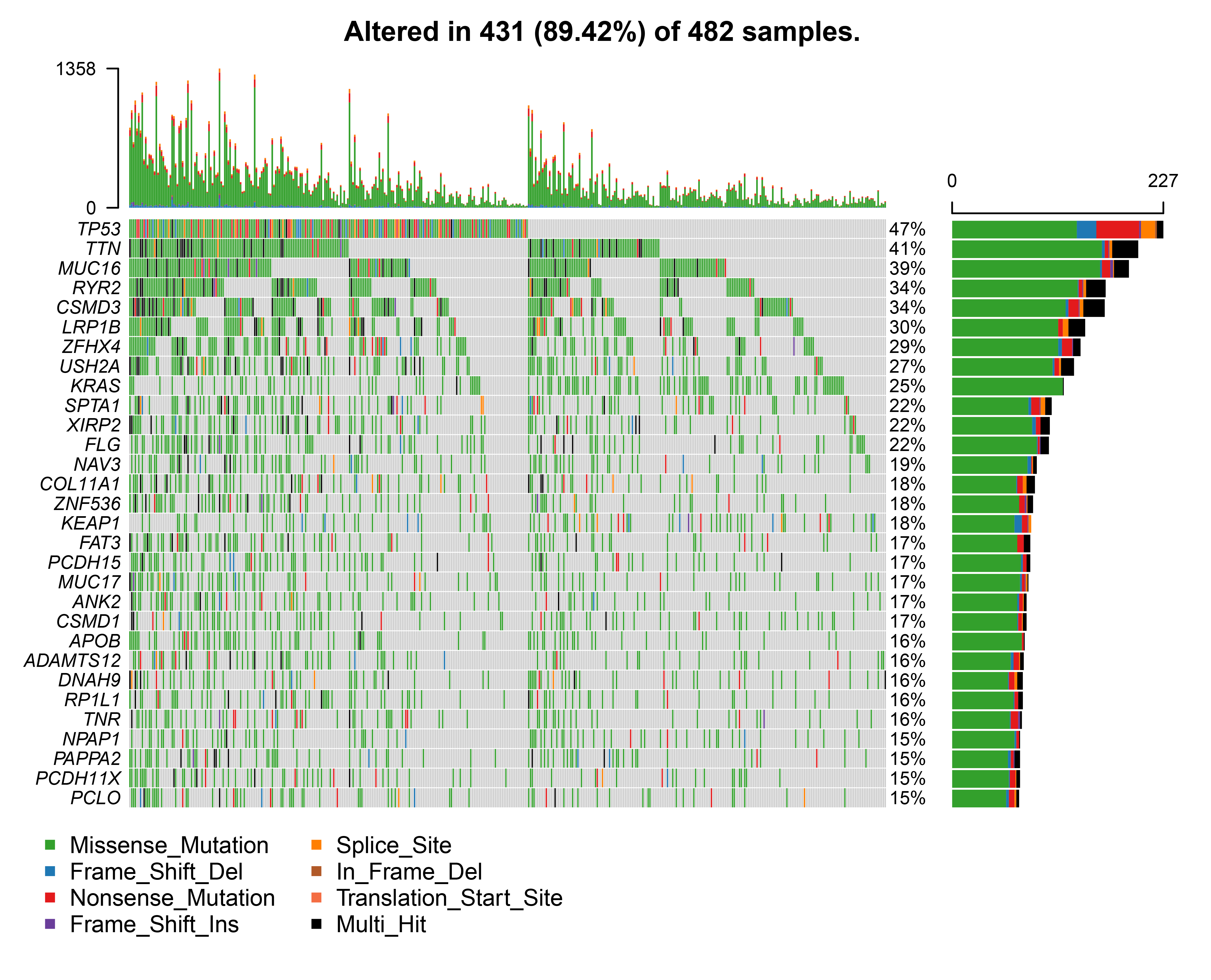

Supplement: Supplementary file 1 [file DataSheet1.zip › Supplementary data/Supplementary Figure S4.tif]
